# Reduced Screen Time is Associated with Healthy Dietary Behaviors but Not Body Weight Status among Polish Adolescents. Report from the Wise Nutrition—Healthy Generation Project

**DOI:** 10.3390/nu12051323

**Published:** 2020-05-06

**Authors:** Joanna Myszkowska-Ryciak, Anna Harton, Ewa Lange, Wacław Laskowski, Agata Wawrzyniak, Jadwiga Hamulka, Danuta Gajewska

**Affiliations:** 1 Department of Dietetics, Institute of Human Nutrition Sciences, Warsaw University of Life Sciences (WULS), 159C Nowoursynowska Str, 02-776 Warsaw, Poland; anna_harton@sggw.pl (A.H.); ewa_lange@sggw.pl (E.L.); danuta_gajewska@sggw.edu.pl (D.G.); 2 Department of Food Market and Consumer Research, Institute of Human Nutrition Sciences, Warsaw University of Life Sciences (WULS), 159C Nowoursynowska Str, 02-776 Warsaw, Poland; waclaw_laskowski@sggw.edu.pl; 3 Department of Human Nutrition, Institute of Human Nutrition Sciences, Warsaw University of Life Sciences (WULS), 159C Nowoursynowska Str, 02-776 Warsaw, Poland; agata_wawrzyniak@sggw.edu.pl (A.W.); jadwiga_hamulka@sggw.edu.pl (J.H.)

**Keywords:** screen time, dietary behaviors, body weight status, adolescents

## Abstract

Screen time (ST) not only affects physical activity but can also be associated with dietary behaviors. Both of these factors determine the health and development of adolescents. The aims of the study were: 1. to analyze the relationship between ST and nutritional behaviors among adolescents; 2. to examine this association in relation to body weight status. Data on the ST duration and nutritional behaviors were collected using a questionnaire. Body mass status was assessed based on weight and height measurements. A total of 14,044 students aged 13–19 years old from 207 schools participated in the study. A significant relationship between ST and gender, age and type of school was observed, but not body weight status. The average ST duration increased with age (from 2.6 h among 13 years old to 3.2 h among 19 years old), and was significantly higher among boys in all age categories (2.7 h vs. 2.5 h in the youngest age group, and 3.5 h vs. 3.0 h in the oldest age group, respectively). The chance for meeting the recommendation for ST in a group of girls (regardless of age) was almost 50% higher compared to boys. Meeting ST recommendation (≤2 h) was associated with a greater odds ratio for favorable nutritional behaviors in the whole group, with exception of drinking milk or milk beverages, and significantly reduced the odds ratio of adverse dietary behaviors (drinking sweet beverages, consumption of sweets and fast food) in the whole group and by gender. More research is needed to clarify the possible cause-and-effect relationships between ST and dietary behaviors.

## 1. Introduction

A key condition for proper growth and development, as well as achieving and maintaining good health is a balanced diet and regular physical activity [1]. This is reflected in the general recommendations of healthy nutrition equally directed to adults, children and adolescents. Such recommendations, presented in the graphic form of a pyramid or a plate, increasingly include physical activity as an integral part of a healthy lifestyle [2], determining the amount of food to be consumed. In the recommendations addressed to the adult population in Poland, physical activity is placed at the base of the food pyramid, in the form of images depicting types of physical activity, (e.g., Nordic walking, cycling or climbing stairs) [3]. In the case of children and adolescents aged 4–18 years old, physical activity is not only positioned at the base of the pyramid, but also listed among the 10 most important principles of a healthy lifestyle. Physical activity, for at least one hour a day, and limiting use of TV, computer and other electronic devices to 2 h are strongly recommended for these age groups [4].

Polish recommendations for children and adolescents regarding screen time (ST), including TV/DVD watching, playing games on a computer or games console, using a computer etc., are in line with the worldwide applied recommendations of The American Academy of Pediatrics (AAP) [5]. However, the World Health Organization (WHO), in its latest recommendations for younger children, suggests greater ST restrictions: up to 1 h per day for children from 2 to 4 years old, while in the younger age group (0–1 years old), no screen time is recommended [6].

Policy on sedentary behaviors, especially screen time, is very important for the health and proper development of the pediatric population. Technological progress, digitization of all areas of life, as well as the increasing availability of electronic equipment, including computers, smartphones, televisions, PlayStations, software and computer/video games, affect children and adolescents behaviors, and cause a prolonged time spent passively, in front of the screen [7,8,9].

In the Czech Republic, the percentage of boys aged 10.5–16.5 exceeding the recommended screen time increased from 75% in 2002 to almost 89% in 2014. For girls, the percentage was lower and amounted to 61% and 77%, respectively [10]. Only 32.3% of American adolescents aged 16–19 recruited in the 2005–2006 National Health and Nutrition Examination Survey (NHANES) met screen time recommendations [11]. In the 13-year-old German cohort, the average self-reported screen time was over 2 h a day [12]. Data for younger age categories are also alarming. Results from the Childhood Obesity Surveillance Initiative (COSI) study of 63,215 children aged 6, 7, 8 and 9 from 19 countries (including Poland) showed that the average daily screen time in Northern European countries was 1.9 h, slightly less (1.7 h) in Eastern European countries and the lowest (1.4 h) in Southern European/Mediterranean Countries [13].

More and more concerns have been growing about the impact of screen time (especially the use of mobile phones), on children and adolescents’ health and well-being. In a systematic review of reviews, Stiglic and Viner [14] found moderately strong evidence for associations between screen time and greater obesity/adiposity and higher depressive symptoms. At the same time, moderate evidence for an association between ST and higher energy intake, less healthy diet quality and poorer quality of life was observed. In the case of behavior problems, such as anxiety, hyperactivity and inattention, poorer self-esteem, well-being and psychosocial health, the evidence for associations with screen time was weak. Although the analysis included observations on populations from 0 to 24 years of age, the conclusions concerned the whole group, without divisions into age categories. The authors pointed out the urgent need for further research to understand the impact of the contexts and content of screen use on the health and well-being of children and adolescents.

Such a rapid “digitization” of children and adolescents’ surroundings causes dramatic changes in their lifestyle. This shift has been observed for a relatively short time—over the past decade. Hence, there is a need for more research combining screen time with other determinants of the lifestyle, to inform policy. Screen time might be associated with different health indicators differently, so their clustering should be independently studied. Moreover, existing relationships can vary depending on age, gender or body weight status. The aims of the study were: 1. to analyze the relationship between screen time and dietary behaviors among adolescents, and 2. to examine this association in relation to the body weight status. To our best knowledge, this is the only observation of such a large and representative group of students aged 13–19 years old linking screen time with dietary behaviors and the body weight status in Poland. Research in this population group is particularly important, because lifestyle behaviors (e.g., unhealthy diet and physical inactivity) are modifiable, and usually established during youth or young adulthood [15]. The transition from childhood to adolescence is associated with a combination of stressors, which can have a significant impact on individuals’ health lifestyle choices [16]. Furthermore, a good health status, especially in relation to normal body weight early in life, reduces the risk of obesity and diseases related to excessive body mass later in life [17].

## 2. Materials and Methods

### 2.1. General Information

These results are part of the Wise Nutrition, Healthy Generation (WNHG) project, granted by The Coca-Cola Foundation and conducted in 2058 schools (attended by nearly 450,000 students) within years 2013–2015. It was an education and research program, free of any charge for all participants, addressed to the secondary and upper secondary school students, their parents and teachers. The educational part included classes for school youth, conducted by trained educators, training and educational materials for teachers and parents of students, covering topics related to a healthy lifestyle, including the role of properly balanced diet and adequate physical activity for health and prevention of diet-related non-communicable chronic diseases. The research part was aimed at assessing eating behaviors, screen time duration and body weight status of Polish adolescents aged 13–19 years old. Students with diagnosed abnormal body weight were invited to the free of charge dietary counseling program (two individual meetings with a dietician). A detailed description of the program (including recruitment procedure) has been described in previously published articles on nutritional behaviors [18] and body weight status of adolescents [19]; the overall scheme of the program is available in the Supplementary Materials (Figure S1).

When designing the program, the standards required by the Helsinki Declaration and the national ethical guidelines were followed. The personal data of participants were fully deidentified. The final protocol of the study was approved by the Scientific Committee of the Polish Society of Dietetics. The program received the patronage of the Minister of National Education and local government educational institutions. Prior to the recruitment, all participants received detailed information about the program (covering its purpose, scope and all procedures) and the possibility of withdrawal at any stage without consequences. The school headmaster provided a written permission to conduct educational activities in the institution, while students’ parents/legal guardians and adolescents over 16 years of age provided written informed consent to participate in the research part of the program, including the questionnaire and anthropometric measurements.

This paper focused on the relationship between screen time and nutritional behaviors among Polish adolescents in relation to the body weight status.

### 2.2. Study Participants

Study participants were recruited from schools participating in the program. To ensure a representative selection of the sample, these schools were randomly selected using the stratified sampling method from the total of 2058 enrolled institutions. The sampling was stratified by: (1) province (based on administrative division); (2) location (large, medium, small city and countryside); (3) the type of school (secondary^1^ and upper secondary^2^). Then, in selected schools, students were randomly drawn from the class registry. The following exclusion criteria were used: health condition requiring the special diet prescribed by a physician, pregnancy or lactation in girls and no written consent. Based on the adopted criteria, 207 schools were selected for the study (~10% of the total number of 2058 institutions), and 14,044 students, including 7553 (53.8%) girls and 6491 (46.2%) boys aged 13–19 years old. The criteria for age groups were adopted according to the Health Behavior in School-Aged Children (HBSC) study protocol [20].

### 2.3. Nutritional Behaviors and Screen Time

Data on nutritional behaviors and the time spent in front of the screen, were collected using pen-and-paper personal interview (PAPI) method. The questionnaire was provided by a dietician to each student individually, prior the education activities and anthropometric measurements. It included questions about the selected nutritional behaviors, the average time spent in front of the screen every day (screen time) and self-satisfaction with body weight (the latter is not included in this article). Explanations were given if necessary.

When developing the questionnaire, the HBSC [21] protocol was adapted with some modification. HBSC is a cross-national study on adolescents’ health behaviors conducted for more than 30 years. A detailed methodology for developing the questionnaire, as well as an information on its validation has been described in the previously published article on adolescents’ nutritional behaviors [18]. The nutritional part of the questionnaire consisted of nine simple questions, requiring “yes” or “no” answers. Six questions referred to positive nutritional behaviors as follows: (1) regular consumption of breakfast before leaving for school; (2) daily consumption of at least one serving of fresh fruit; (3) daily consumption of at least two servings of vegetables; (4) daily consumption of milk and/or milk fermented beverages; (5) daily consumption of whole grains; (6) consumption of fish at least once a week. Three questions concerned the adverse nutritional practices as: (1) drinking sugared soft drinks (soda and other carbonated soft drinks) several times during the week; (2) eating sweets more than once a day; (3) consuming fast food more than twice a week.

In addition, participants were asked about the time spent passively in front of a computer screen, TV screen, etc. during a typical day (average number of hours/day), and their satisfaction with the actual body weight (the latter data is not discussed in this article). In the assessment of screen time behaviors, the value of 2 h was used as the cut-off point, in accordance with the recommendations of the American Academy of Pediatrics (AAP) [5] and the Polish Institute of Food and Nutrition [4]. Values up to 2 h were found to be in line with the recommendations.

### 2.4. Anthropometric Measurements

Anthropometric measurements, including the body weight and height of individuals, were conducted by specially trained dietician, following standardized procedure according to the Anthropometry Procedures Manual by National Health and Nutrition Examination Survey (NHANES) [22]. All the measurements were carried out with the equipment provided by The Polish Society of Dietetics: digital floor scales (TANITA HD-380 BK, Tanita Corporation, Tokyo, Japan) and steel measuring tapes (0–200 cm) to minimize the bias. The school provided a room suitable for the measurements. Detailed description of all the procedures is presented in previously published papers [18,19].

The assessment of the body weight status of students was based on body mass index (BMI, weight in kg/height in m^2^). For students aged 18 years old and younger, calculated BMI value was plotted on gender BMI centile charts for age (with an accuracy of one month) [23], and the percentile value was read from percentile grids. As recommended by the International Obesity Task Force (IOTF) cut-offs, the sample was classified into four body mass categories according to percentiles: below the 5th percentile was underweight, between the 5th and 84th was normal weight, between the 85th and 94th was overweight and the 95th percentile and above was obese [24]. The WHO standard BMI criteria were applied for individuals above the age of 18 years old as follow: BMI <18.5 kg/m^2^—underweight; BMI 18.5–24.9 kg/m^2^—normal body weight; BMI 25–29.9 kg/m^2^—overweight; BMI ≥ 30 kg/m^2^—obesity [25].

### 2.5. Statistical Analysis

Descriptive statistics and distribution normality testing of the continuous variables were performed with the Shapiro-Wilk test; the results were presented as means, standard deviations and as percentages according to the type of variable. Data were analyzed in the total group, according to age, gender and body weight status. Statistical significances for nominal variables were determined using the Pearson’s chi-square test. Additionally, contingency coefficient Cramer’s V was used to indicate the strength of association between categorical variables. For non-normally distributed variables, the Mann-Whitney U test was used. The correspondence analysis and odds ratio were used to study the relationship between screen time duration and selected indicators (age, type of school, body weight status, dietary behaviors). For all tests, *p* < 0.05 was considered as significant. All statistical analyses were conducted using Statistica version 13.1 (Copyright^©^StatSoft, Inc, 1984–2014, Cracow, Poland).

## 3. Results

A total of 14,044 adolescents (53.8% girls and 46.2% boys), 13 to 19 years old, participated in the study. Descriptive statistics for the sample are presented in Table 1. Significant differences were observed for body weight, height and BMI values between girls and boys in all age categories, except for body mass index for the group of 13-year-olds. The average screen time increased with age and was significantly higher among boys in all age categories.

The characteristics of the group in terms of gender, age, type of school and weight status, including the screen time criterion, are presented in Table 2. A significant impact of gender, age and type of school on screen time was observed. The probability of meeting the recommended screen time was almost 50% higher among girls compared to boys. With the youngest age group as a reference, the odds of not exceeding the screen time recommendations decreased from about 80% among 14-year-olds, to less than 60% in the oldest group. Secondary school students had almost a 20% greater possibility of meeting the screen time criterion compared with upper secondary students. No significant relationship was observed between the body weight status and screen time duration. Similarly, the body weight status did not significantly affect the odds of meeting the screen time recommendation.

A significant relationship between screen time duration and the majority of analyzed eating behaviors was observed. There was no relationship between ST and drinking milk or milk beverages every day among girls and consuming whole-grained bread every day among boys (Table 3). Meeting the screen time recommendation was associated with a greater odds ratio of favorable nutritional behaviors in the whole group, with exception of drinking milk or milk beverages. Girls who spent up to 2 h in front of the screen were more likely to have regular breakfast, consume fresh fruit, vegetables and fish. Compliance with screen time recommendations was associated with a higher probability of eating breakfast, fresh fruit and fish among boys. Adherence to screen time recommendations significantly reduced the odds ratio of adverse dietary behaviors, such as drinking sweet beverages, consumption of sweets and fast food, in the whole group and by gender.

**Figure 1:**
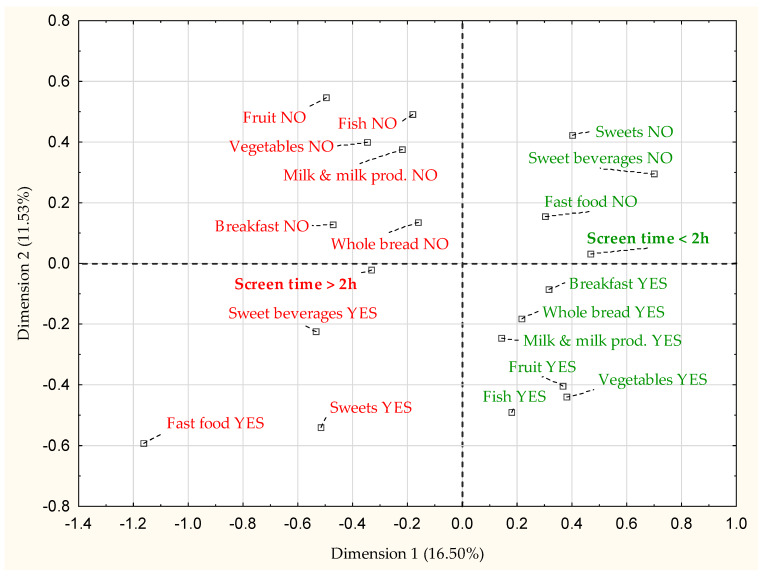
The results of the analysis of correspondence of nutritional behaviors and screen time duration in the total study group (*n* = 14,044). Beneficial behaviors are marked in green; unfavorable behaviors are marked in red.

Figure 1 presents the relationship between the screen time duration within and above the recommendations (0–2 h and > 2 h, respectively), and examined nutritional behaviors in the total group. Based on the correspondence analysis, it was possible to indicate the connections between the analyzed indicators. Beneficial nutritional behaviors, such as consuming breakfast, fruit, vegetables, whole-grain bread, milk or milk beverages and fish were linked together. Whereas unfavorable eating behaviors, such as skipping breakfast, low consumption of milk products, fruits, vegetables, fish and whole-grain bread, were related to each other. Compliance with the recommended screen time corresponded to the beneficial eating behaviors, such as avoiding sweets, sweet beverages, fast food and consuming breakfast, whole-bread and milk and milk beverages. Exceeding screen time recommendation was more associated with adverse nutritional behaviors, i.e., skipping breakfast, drinking sweet beverages and avoiding whole-grained bread.

## 4. Discussion

Recently, more and more attention is focused not on the individual lifestyle factors (e.g., physical activity, fruit and vegetable consumption, etc.), but on the complex/cluster analyses of energy balance-related behaviors [26,27,28]. However, the majority of research on screen time is focused on the relationship with the body mass index or other indicators of nutritional status, e.g., percentage of body fat [29,30,31,32]. Our study shows the relationship between screen time and various dietary behaviors, important indicators of diet quality, in a large sample of adolescents aged 13–19. Indication of co-existing effects might help to understand which behaviors should be approached simultaneously in health-promoting actions.

The main observations of the study revealed that: (1) the average screen time in all age groups exceeded the recommendations, it increased with age and was higher among boys; (2) reduced screen time (duration within the recommendation) was associated with a higher odds ratio of favorable dietary behaviors, and at the same time a lower probability of adverse dietary behaviors; and (3) screen time duration below the recommendation corresponded to beneficial dietary behaviors, for screen time above the guidelines, the opposite relationships were observed.

### 4.1. Screen Time Behaviors

Recent research indicates that screen time recommendations are exceeded by 40% to 80% of children and adolescents [33,34] in Western populations. Over a third of Chinese junior high and senior high school students failed to meet the guidelines [35]. Our study confirms the problem of excessive screen time among 58.6% of Polish students. Moreover, we observed a significant impact of gender and age (as the type of school is related to the age of students) on adherence to the recommendation. The chance for meeting the recommendation for screen time in a group of girls (regardless of age) was almost 50% higher compared to boys. At the same time, the chance to comply with screen time guidelines among 19-year-olds was over 40% lower compared to 13-year-old children. Yan et al. [35] also observed more frequent occurrence of exceeded screen time among boys. Therefore, our findings put into question previous observations [36,37] about the greater physical activity of boys compared to girls, and show the need for gender focused educational activities. However, the observed effect of gender on the screen time duration requires further investigation. The observed increase in unfavorable screen time behaviors with age indicates the need for systematic educational activities from an early age to overcome the unfavorable trend. Especially, the observations of younger children from six European countries (including Poland) showed that about 70% of preschoolers exceed their screen limit [38]. Furthermore, there are evidence that screen time behaviors may form in youth [39].

### 4.2. Screen Time and Body Weight Status

In our study, we found no relationship between the screen time duration and body weight status. Theoretically, screen time belongs to sedentary behaviors, characterized by a low energy cost [40], which can decrease the total energy expenditure, and consequently promote weight gain. Sedentary behaviors, which are associated with activities with a very low energy expenditure (1.0–1.8 metabolic equivalent), performed mainly in a sitting or lying position, were related to an increase in obesity [41,42]. The results of numerous studies showed a positive correlation between screen time duration and body weight in different population groups [43,44,45]. A recent systematic review and meta-analysis of 16 studies showed a positive association between the different types of screen time and overweight/obesity among children and adolescents (< 18 years) [46]. However, this relationship is not so obvious, as there is a hypothesis that sedentary behaviors (e.g., screen time) can be compensated for with intense physical activity during the rest of the time. Sevil-Serrano et al. [27] analyzed health-related behaviors (including screen time, physical activity, healthy diet and sleep duration) and body mass index among 173 Spanish adolescents. They observed an equal distribution of sedentary screen time in the different profiles of other behaviors, but no association with the body weight status was found. The meta-analysis on 30 studies, with a total of 44,707 subjects under 18 years of age, published since 1985, found a small, significant positive relationship between television viewing and body fatness, but no association between body fatness and video game or computer use [47]. The type of activity performed may be of key importance for assessing the relationship between screen time and eating behavior. For example, a higher proportion of TV viewing in the total screen time may be associated with the impact of commercial for foods rich in fat, sucrose and salt, promoting greater consumption of these products [48]. Further research seems to be needed on the impact of screen time (by type) on body weight and the risk of excessive body mass among adolescents.

### 4.3. Screen Time and Dietary Behaviors

Screen time, as a sedentary activity, might affect both the amount, and quality of food consumed while staying inactive. The unfavorable associations between screen time during the meals, total daily screen time and junk food consumption was reported by Jusiene et al. [49] in Lithuanian children aged 2 to 5 years old. A cross-sectional analysis of 630 Canadian children aged 8–10 years old showed that prolonged screen time was associated with a higher intake of energy, lower intake of fiber, vegetables and fruit [50]. Longer TV viewing time was correlated with a lower protein, calcium, vitamin B_2_, and total dietary fiber (in boys) intake in Japanese children and adolescents [51]. Young adolescents with longer screen time (> 6 h) were less likely to have a good overall diet quality compared with their peers, with less than one hour of afterschool–evening screen time. In this study increased evening screen time was linked to fewer evening snack servings of fruit and vegetables [52]. There is also evidence that sedentary behaviors (screen time and TV viewing) in children, adolescents and adults appear to be associated with selected indicators of a less healthy diet including: lower fruit and vegetable consumption, higher consumption of energy-dense snacks, drinks and fast foods [53]. However, to our knowledge, none of the earlier studies analyzed many indicators of diet quality simultaneously, in such a wide age range of a large and representative population. Our study confirmed the relationship between screen time and diet quality: in the whole group, significant relationships were noted for all examined factors (eating breakfast, consuming fresh fruit vegetables, milk and milk beverages, whole-grain bread and fish, and avoiding sweet beverages, sweets and fast-foods). The relationships for the total group remained the same for both sexes, except for drinking milk and milk beverages among girls and consuming whole grain bread among boys. Adolescents adhering to the screen time recommendation were more likely to demonstrate healthy eating behaviors, such as: having regular breakfast, consuming fresh fruit, vegetables, whole-grain bread, fish and avoiding adverse dietary behaviors, as: drinking sweet beverages, consuming sweets and fast-foods. In this case, slight gender differences were observed: compliance to the screen time recommendations did not increase the chance of eating whole-grain bread among girls and boys, nor vegetables among boys. It can be assumed that screening time can have a greater impact on the eating behavior than gender. However, significant differences in diet quality between females and males have been previously reported in adolescents. Gaylis et al. [54] showed that girls consumed vegetables and fruit more frequently than boys, where boys consumed fast-food, fruit juice and soda more frequently than females.

In our study, the relationship between the screen time and dietary behaviors is confirmed by correspondence analysis: adherence to recommended screen time corresponded to eating breakfast and avoiding fast-food. On the other hand, screen time above guidelines corresponded to adverse dietary behaviors: drinking sweet beverages and avoiding whole grain bread and regular breakfasts. This indicates the need for a comprehensive approach to educational activities, as none of the dietary behaviors should be treated separately to achieve a significant improvement in overall diet quality. This may also contradict the hypothesis that negative eating behaviors can occur simultaneously with positive ones (e.g., drinking sweet beverages but eating vegetables or fruits at the same time). Further studies are needed to clarify the possible relationship between different dietary behaviors and dietary behaviors and screen time.

### 4.4. Study Strengths and Limitations

An important strength of this study is a large and random sample size and the use of valid and reliable measures to assess the study variables (e.g., criteria for assessing the body weight status). To our knowledge, there is no nationwide research covering all age categories of adolescence in Poland. Furthermore, the body weight status was assessed based on the data obtained through measurements, not self-reported data of body height and weight, which is common procedure in large studies [21]. All anthropometric measurements were conducted with a standardized procedure by specially trained dieticians, which ensured obtaining reliable results and minimized the bias.

Our study is subject to a number of limitations. Firstly, we did not examine overall sedentary behaviors: screen time was analyzed separately from other forms of sedentary behavior (e.g., sitting while talking, homework, reading, time spent in a car etc.). However, according to Lee et al. [29], screen time was more important for anthropometric parameters and body mass index compared to other sedentary activities among a representative sample of children. Secondly, the screen time duration and dietary behaviors were self-reported. However, the presence of a trained interviewer when completing the questionnaire gave the respondents opportunity to clarify possible doubts, and reduced the possibility of misunderstanding the question. Thirdly, as our study is cross-sectional, we cannot yet conclusively determine the directional effects. For example, whether extended screen time resulted in poorer dietary habits, or whether a less correct diet favored less pro-healthy sedentary behaviors like extended screen time.

## 5. Conclusions

The present study adds new data on screen time and adolescent’s nutritional behaviors in two important aspects. Firstly, we confirmed the unfavorable associations between daily screen time and less healthy dietary habits in a large, representative group of Polish adolescents aged 13–19 years old. Secondly, the results of our study did not confirm the relationship between the screen time and body weight status. In order to create effective health-promoting programs, more research is needed to clarify the possible cause-and-effect relationships between dietary behavior and screen time.

## Figures and Tables

**Table 1:** Descriptive statistics: anthropometric data and the average screen time duration (all data expressed as mean ± SD).

Age [years]	Height [cm]		Weight [kg]		BMI [kg/m^2^]		Screen Time [h]	
	Girls	Boys	Girls	Boys	Girls	Boys	Girls	Boys
13 14 15 16 17 18 19	160.8 ± 6.5 ^a^ 163.0 ± 5.9 ^a^ 163.9 ± 5.9 ^a^ 164.9 ± 6.1 ^a^ 164.9 ± 5.9 ^a^ 165.2 ± 5.8 ^a^ 165.3 ± 6.5 ^a^	163.4 ± 8.6 ^b^ 169.3 ± 8.5 ^b^ 173.8 ± 3.4 ^b^ 176.8 ± 6.8 ^b^ 178.3 ± 6.7 ^b^ 178.9 ± 6.8 ^b^ 179.4 ± 6.8 ^b^	51.6 ± 10.9 ^a^ 54.1 ± 10.3 ^a^ 55.5 ± 9.6 ^a^ 57.4 ± 9.2 ^a^ 57.9 ± 9.5 ^a^ 58.1 ± 9.4 ^a^ 59.6 ± 11.2 ^a^	54.5± 12.8 ^b^ 59.5 ± 12.9 ^b^ 63.7 ± 12.6 ^b^ 67.7 ± 12.7 ^b^ 71.1 ± 12.8 ^b^ 72.8 ± 12.7 ^b^ 73.0 ± 12.7 ^b^	19.9 ± 3.520.3 ± 3.4 ^A^ 20.6 ± 3.2 ^A^ 21.1 ± 3.1 ^a^ 21.3 ± 3.3 ^a^ 21.3 ± 3.1 ^a^ 21.7 ± 3.7 ^a^	20.3 ± 3.820.6 ± 3.5 ^B^ 21.1 ± 3.5 ^B^ 21.6 ± 3.5 ^b^ 22.3 ± 3.6 ^b^ 22.7 ± 5.3 ^b^ 22.6 ± 3.5 ^b^	2.5 ± 1.5 ^A^ 2.7 ± 1.5 ^a^ 2.8 ± 1.5 ^a^ 2.8 ± 1.5 ^a^ 2.8 ± 1.6 ^a^ 2.8 ± 1.5 ^a^ 3.0 ± 1.7 ^a^	2.7 ± 1.6 ^B^ 3.0 ± 1.6 ^b^ 3.2 ± 1.8 ^b^ 3.1 ± 1.6 ^b^ 3.2 ± 1.8 ^b^ 3.4 ± 1.8 ^b^ 3.5 ± 2.0 ^b^

^A,B^ significant differences between girls and boys, the Mann-Whitney *U* test, *p* < 0.05; ^a,b^ significant differences between girls and boys, the Mann-Whitney *U* test, *p* < 0.001.

**Table 2:** Characteristics of the study group (*n* = 14,044), depending on the screen time (ST) duration and the odds ratio (OR; CI, confidence interval) for screen time duration within the recommendations (≤ 2 h) depending of analyzed factors.

Factor	Total [%]	ST ≤ 2 h [%]	ST > 2 h [%]	*p*-Value * /V Cramer	OR for ST ≤ 2 h [95% CI]
Sex				< 0.001 */0.10	
Girls *n* = 7553	53.8	59.6	49.6		1.499 (1.401–1.605) **
Boys *n* = 6491	46.2	40.4	50.4		0.667 (0.623–0.714) **
Age [years]				< 0.001 */0.07	
13	9.0	11.1	7.6		1.0 (reference)
14	13.3	14.8	12.3		0.820 (0.711–0.946) **
15	14.3	13.7	14.7		0.639 (0.554–0.736) **
16	17.0	16.4	17.4		0.643 (0.561–0.738) **
17	19.8	19.3	20.2		0.654 (0.573–0.748) **
18	18.2	17.2	18.9		0.623 (0.544–0.714) **
19	8.3	7.5	8.9		0.575 (0.489–0.676) **
Type of school				< 0.001 */0.04	
secondary	42.1	44.5	40.4		1.185 (1.107–1.269) **
upper secondary	57.9	55.5	59.6		0.844 (0.788–0.903) **
Body weight status				0.680	
underweight	5.1	4.9	5.2		0.926 (0.793–1.082)
normal	76.7	77.0	76.4		1.0 (reference)
overweight	11.6	11.4	11.8		0.959 (0.863–1.067)
obesity	6.6	6.7	6.6		1.011 (0.882–1.158)

* significant differences between screen time ≤ 2 h and screen time > 2 h, the Pearson’s chi-square test; ** significant differences *p* < 0.001, the Wald test.

**Table 3:** Nutritional behaviors of the individuals (*n* = 14,044) depending on screen time (ST) duration and the logistic regression analyses for the association of meeting ST recommendations (≤ 2 h) on nutritional behaviors (OR, odds ratio; CI, confidence interval).

Factor	ST ≤ 2 h [%]	ST > 2 h [%]	*p*-Value * /V Cramer	OR for ST ≤ 2 h [95% CI]
**Having breakfast every day before leaving for school**	63.7	57.1	< 0.001 */0.06	1.197 (1.115–1.285) **
Girls, *n* = 7553	55.9	44.9	< 0.001 */0.08	1.253 (1.140–1.377) **
Boys, *n* = 6491	44.1	55.1	< 0.001 */0.07	1.247 (1.116–1.393) **
**Consuming fresh fruit every day (at least 1 serving)**	62.1	54.2	< 0.001 */0.08	1.269 (1.183–1.363) **
Girls	60.2	52.3	< 0.001 */0.06	1.133 (1.029–1.247) ***
Boys	39.8	47.7	< 0.001*/0.10	1.397 (1.256–1.554) **
**Consuming vegetables every day (at least 2 servings)**	50.1	45.7	< 0.001 */0.04	1.073 (1.001–1.150) ***
Girls	60.8	50.1	< 0.001 */0.05	1.105 (1.005–1.213) ***
Boys	39.2	50.0	0.009 */0.03	1.034 (0.932–1.148)
**Drinking milk or milk beverages every day**	61.3	59.7	0.047 */0.02	1.021 (0.952–1.096)
Girls	54.9	45.1	0.052	1.041 (0.949–1.143)
Boys	45.1	54.9	0.004 */0.03	1.102 (0.987–1.231)
**Consuming whole-grained bread every day**	44.3	41.1	0.002 */0.03	1.078 (1.006–1.156) ***
Girls	61.2	50.9	0.004 */0.03	1.056 (0.962–1.160)
Boys	38.8	49.1	0.052	1.083 (0.975–1.202)
**Consuming fish at least once a week**	52.7	48.1	< 0.001 */0.04	1.162 (1.085–1.245) **
Girls	53.9	44.4	< 0.001 */0.04	1.163 (1.060–1.277) ***
Boys	46.1	55.5	< 0.001 */0.07	1.275 (1.148–1.416) **
**Drinking sweet beverages few times a week**	49.8	61.7	< 0.001 */0.12	0.702 (0.653–0.754) **
Girls	52.0	44.4	< 0.001 */0.10	0.729 (0.662–0.804) **
Boys	48.0	55.6	< 0.001 */0.09	0.763 (0.684–0.852) **
**Consuming sweets more than once a day**	38.6	47.5	< 0.001 */0.09	0.773 (0.720–0.829) **
Girls	61.7	52.2	< 0.001 */0.10	0.743 (0.676–0.818) **
Boys	38.3	47.8	< 0.001*/0.08	0.754 (0.677–0.840) **
**Consuming fast food more than 2 times a week**	16.6	23.6	< 0.001 */0.08	0.799 (0.730–0.875) **
Girls	52.6	43.9	< 0.001 */0.08	0.824 (0.725–0.936) ***
Boys	47.4	56.1	< 0.001 */0.08	0.818 (0.718–0.932) ***

* significant differences between screen time ≤ 2 h and screen time, the Pearson’s chi-square test; ** significant differences *p* < 0.001, the Wald test; *** significant differences *p* < 0.05, the Wald test.

## Footnotes

1 The second stage of education after primary school, compulsory for all adolescents aged 13–16 years in Poland.

2 The next stage of education includes high schools, technical schools and vocational schools for adolescents aged 16–20 years old.

## Supplementary Materials

The following are available online at https://www.mdpi.com/2072-6643/12/5/1323/s1, Figure S1: The Wise Nutrition—Healthy Generation project diagram.
