# Supplementary material for: Reduced Screen Time is Associated with Healthy Dietary Behaviors but Not Body Weight Status among Polish Adolescents. Report from the Wise Nutrition—Healthy Generation Project
Source: Nutrients. 2020 May 6;12(5):1323. doi: 10.3390/nu12051323 (PMC7285006; doi:10.3390/nu12051323)
Supplement: Supplementary file 1 [file nutrients-12-01323-s001.pdf]

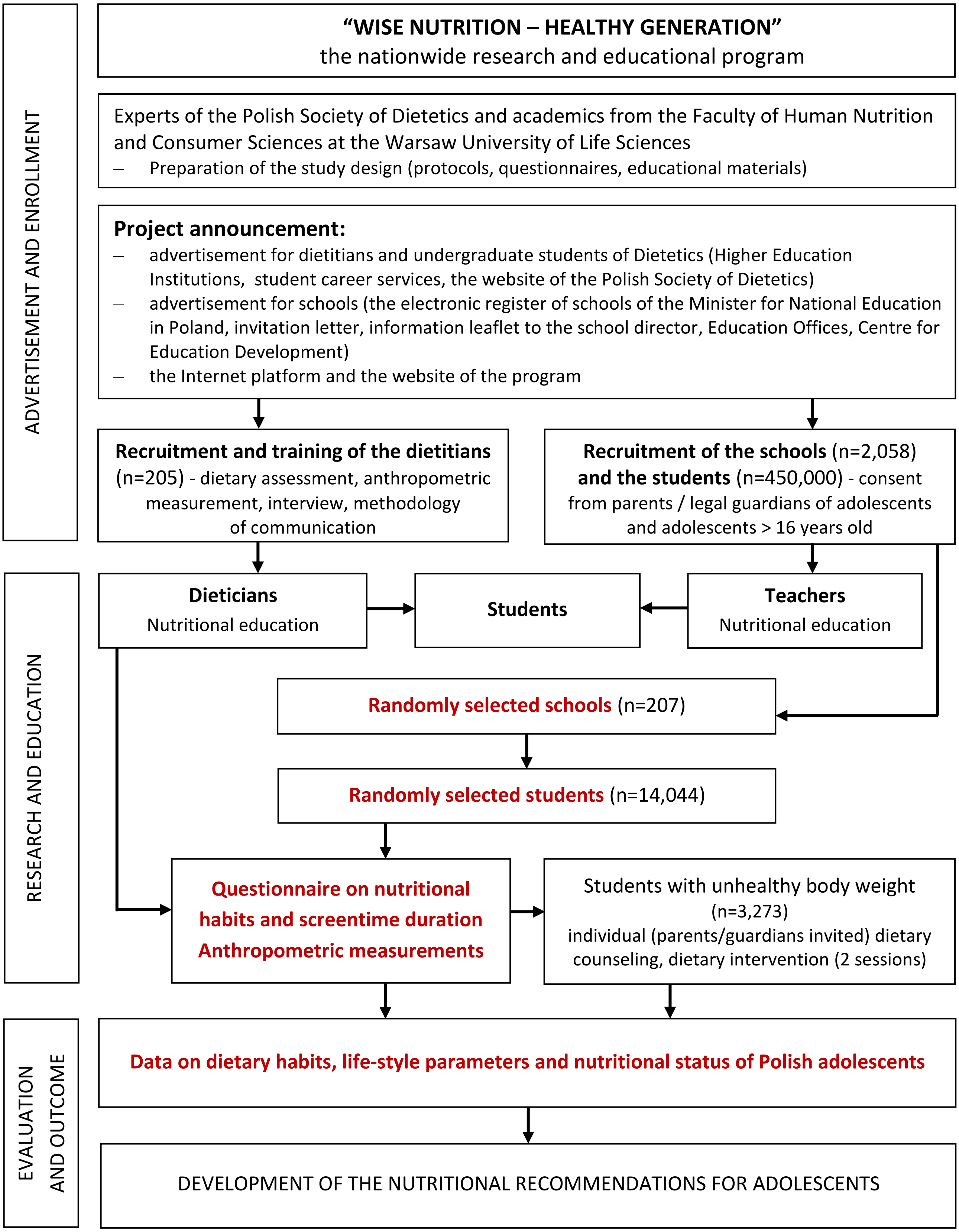

Figure 1. The Wise Nutrition - Healthy Generation project diagram (the part of the program presented in the article is marked in red).
